# Mechanical Load‐Induced Upregulation of Talin2 through Non‐Canonical Deubiquitination of OTUB1 Drives Facet Joint Osteoarthritis Pathogenesis

**DOI:** 10.1002/advs.202501046

**Published:** 2025-04-25

**Authors:** Yizhen Huang, Heng Sun, Haojie Chen, Xiangpeng Wang, Junduo Zhao, Yang Jiao, Hongyi Zhou, Haoyu Cai, Jiafeng Dai, Xuan Huang, Weiyun Chen, Jianxiong Shen

**Affiliations:** ^1^ Department of Orthopaedics Peking Union Medical College Hospital Peking Union Medical College and Chinese Academy of Medical Sciences Beijing 100730 P. R. China; ^2^ Graduate School of Peking Union Medical College and Chinese Academy of Medical Sciences Beijing 100730 P. R. China; ^3^ Department of Rheumatology and Clinical Immunology Peking Union Medical College Hospital Peking Union Medical College and Chinese Academy of Medical Sciences Beijing 100730 P. R. China; ^4^ Department of Anesthesiology Peking Union Medical College Hospital Peking Union Medical College and Chinese Academy of Medical Sciences Beijing 100730 P. R. China

**Keywords:** extracellular matrix, facet joint osteoarthritis, mechanical loads, non‐canonical deubiquitination

## Abstract

Facet joint osteoarthritis (FJOA) is a prevalent degenerative condition in the aging population; however, the underlying pathophysiological mechanisms remain poorly understood and current therapeutic strategies remain limited to palliative pain management. In this study, novel potential therapeutic targets and prevention paradigms for FJOA are systematically explored. Proteomic screening and validation show that Talin2 is specifically upregulated in FJOA samples. Immunoprecipitation‐mass spectrometry, transcriptome RNA sequencing, and bioinformatics simulation analyses, combined with in vitro and in vivo experiments, are conducted to elucidate the molecular mechanism of the role of Talin2 in FJOA. Increased expression levels of Talin2 in FJOA promote the degradation of the extracellular matrix and inhibit its synthesis. Talin2 is found to be stabilized via non‐canonical deubiquitination and direct interaction with ovarian tumor domain‐containing ubiquitin aldehyde‐binding protein 1 (OTUB1). C–C motif ligand 2 (*CCL2*), an inflammatory chemoattractant, is identified to be a target gene of Talin2. Furthermore, mechanical loading potentiates the Talin2/OTUB1 interaction, resulting in the stabilization of Talin2 and enhances non‐canonical deubiquitination. Therefore, Talin2 regulates *CCL2* expression and promotes FJOA. Given that Talin2 is stabilized and deubiquitinated by OTUB1, especially under mechanical load, the Talin2/OTUB1 interaction may be a promising therapeutic target for FJOA.

## Graphical abstract

Facet joint osteoarthritis (FJOA), prevalent in the elderly, involves Talin2 upregulation promoting ECM degradation while suppressing synthesis. Mechanical load stabilizes Talin2 via OTUB1‐mediated non‐canonical deubiquitination, driving *CCL2* expression that recruits immune cells, exacerbating inflammatory joint degeneration. The role of the Talin2/OTUB1/*CCL2* axis in mechanical load‐inflammatory conversion, suggesting novel potential preventive and therapeutic strategies for FJOA is revealed.

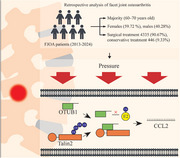

## 1 Introduction

Facet joint osteoarthritis (FJOA), the most prevalent degenerative spinal disorder, is a primary cause of chronic low back pain in the elderly populations, with prevalence rates of 59.6% in males and 66.7% in females. Epidemiological data reveal peak incidence among individuals aged 60–69 years.^[^ ^1^ ^]^ Current therapeutic strategies are limited to pharmacological management (acetaminophen, nonsteroidal anti‐inflammatory drugs, skeletal muscle relaxants, and antidepressants) and minimally invasive interventions, including intra‐articular corticosteroid injections, radiofrequency neurolysis of medial branches, and cryoablation techniques.^[^ ^2^ ^]^ The pathological progression of FJOA manifests as osteophyte formation in facet joints, accompanied by ligamentum flavum hypertrophy, which contributes to the development of lumbar spinal stenosis.^[^ ^3^ ^]^ Despite its clinical significance, FJOA remains substantially underrecognized as a public health burden, necessitating greater emphasis on prophylactic interventions rather than solely targeted symptomatic treatments.

Facet joints serve critical biomechanical functions, facilitating axial load transmission while providing rotational and flexion‐extension stability.^[^ ^4^ ^]^ Chronic biomechanical overloading of the lumbar spine is a well‐established accelerant of spinal degeneration, particularly through load redistribution during intervertebral disc degeneration, which elevates facet joint stresses, accelerating FJOA progression.^[^ ^5^ ^]^ These pathomechanical observations strongly suggest that chronic mechanical overloading is a principal pathogenic mechanism in FJOA development, warranting further investigation into the intrinsic molecular mechanisms.

Proteomic profiling revealed a significant upregulation of Talin2 in FJOA specimens in comparison to controls. Talin2 is a 270 kDa protein that is structurally similar to Talin1 (74% amino acid sequence identity) but functionally different in the mode of integrin activation and bridging interactions with the extracellular matrix (ECM) and cytoskeleton.^[^ ^6^ ^]^ Talin2 interacts with integrins, which promotes invadopodium‐mediated matrix degradation and traction force generation during cancer cell invasion.^[^ ^7^ ^]^ Talin2 comprises an N‐terminal head, which contains an atypical FERM domain that binds β‐integrin tails, and a C‐terminal rod, which has vinculin‐ and actin‐binding sites.^[^ ^8^ ^]^ The binding sites in the C‐terminal rod are exposed through a force‐activated α‐helix swapping mechanism to enhance vinculin recruitment.^[^ ^9^ ^]^ The mechanosensing properties of Talin2 stem from its unique structure, which adopts different conformations to support different signaling pathways and shows switch‐like behavior at different force thresholds for precise mechanical responses.^[^ ^10^ ^]^

Immunoprecipitation‐mass spectrometry was employed to systematically characterize the Talin2 interactome, enabling precise identification of its binding partners. Ovarian tumor domain‐containing ubiquitin aldehyde‐binding protein 1 (OTUB1), belonging to the ovarian tumor domain protease (OTU) subfamily, is a deubiquitinase that blocks ubiquitination.^[^ ^11^ ^]^ Ubiquitination, an enzymatic cascade that occurs in the form of mono‐or polyubiquitination, is important for cellular homeostasis.^[^ ^12^ ^]^ As an OTU‐domain deubiquitinase, OTUB1 directly inhibits ubiquitination through its deubiquitinase activity or binds to ubiquitin‐conjugated E2 via an N‐terminal alpha‐helical extension to prevent ubiquitin transfer.^[^ ^13^ ^]^ In our study, K1543, predicted by PhosphoSitePlus, was not the only ubiquitination site in Talin2, indicating that OTUB1 performs non‐canonical deubiquitination. Whereas the OTUB1^C91A^ mutant retained its ability to suppress ubiquitination, the OTUB1^D88A^ mutation completely abolished this inhibitory function, demonstrating that Asp88 (D88) serves as a functional site. During non‐canonical deubiquitination, OTUB1 preferentially binds to ubiquitin‐conjugated E2, which restricts ubiquitin transfer.^[^ ^14^ ^]^ Moreover, the binding surface of OTUB1/E2 interferes with the interaction between E2 and E3, which further inhibits ubiquitination.^[^ ^15^ ^]^

Following the knockdown of Talin2 expression, transcriptome RNA sequencing was performed to systematically identify downstream regulatory targets. Chemokine C‐C motif ligand 2 (CCL2) is an inflammatory chemoattractant belonging to the CC chemokine superfamily encoded by the *CCL2* gene located on chromosome 17q11.2.^[^ ^16^ ^]^ In rheumatoid arthritis, monocytes are recruited to the synovium by the interaction of CCL2 with chemokine receptor CCR2 and differentiate into macrophages and osteoclasts.^[^ ^17^ ^]^ In addition, the activation of the CCL2/CCR2 signaling has been shown to recruit macrophages, exacerbate synovitis, and damage cartilage in mouse osteoarthritis.^[^ ^18^ ^]^

The present study demonstrates that Talin2, which is upregulated in FJOA cartilage tissues, interacts directly with OTUB1 and undergoes OTUB1‐mediated non‐canonical deubiquitination. Talin2 facilitates the upregulation of *CCL2*, thereby accelerating FJOA pathogenesis. Mechanical loading potentiates Talin2/OTUB1 protein binding, establishing a novel mechanobiological pathway through which mechanical overload exacerbates inflammatory joint degeneration.

## 2 Results

### 2.1 Increased Expression of Talin2 in FJOA is Related to the Biological Functions of Chondrocytes

**Figure 1:**
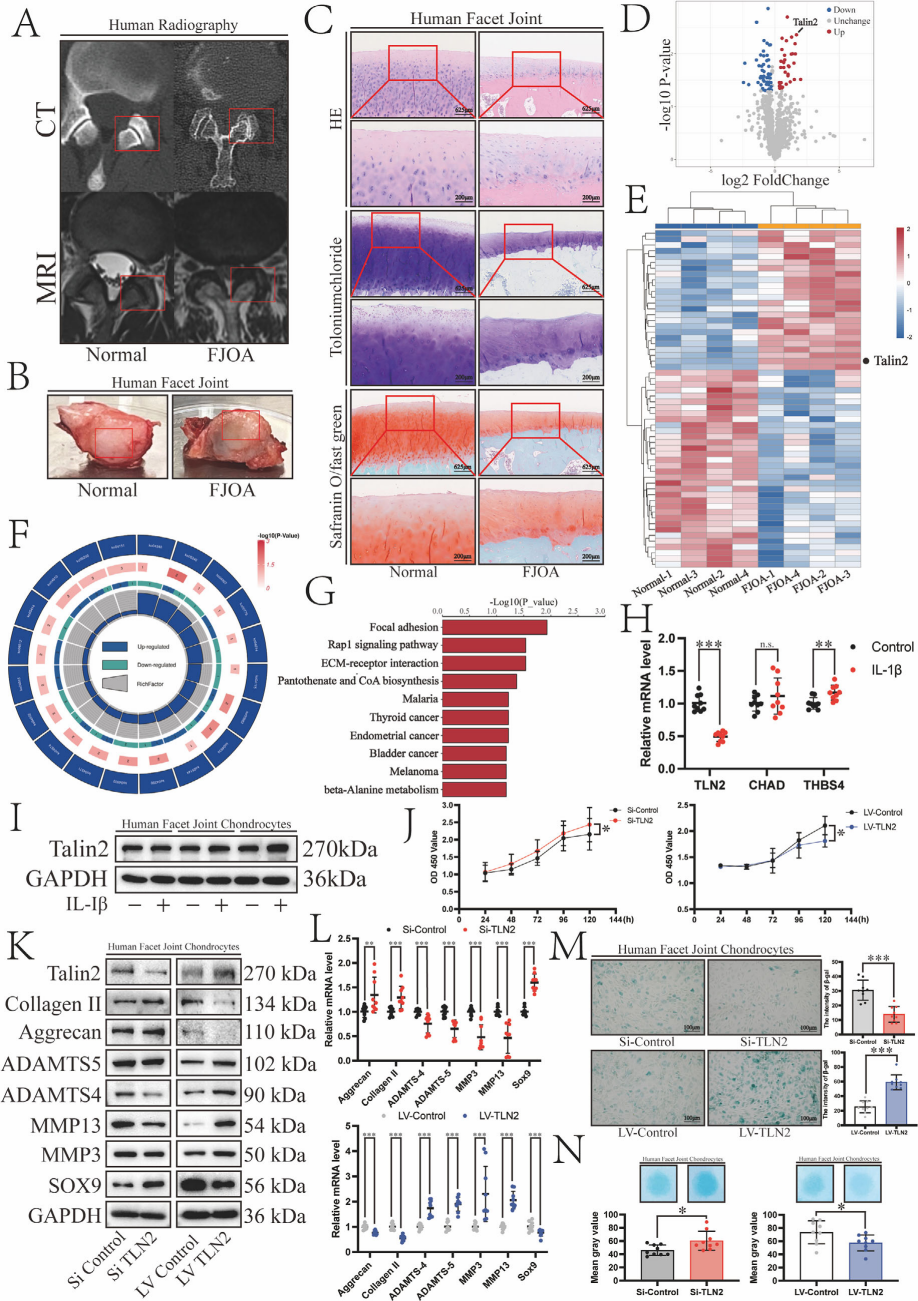
Increased expression of Talin2 in facet joint osteoarthritis (FJOA) is related to the biological function of chondrocytes. A) Computed tomography and magnetic resonance imaging scans of normal and degenerative spines in the transverse plane are presented. The red box marks the facet joint orientation. B) Facet joints of normal and degenerative spines. C) Structure of facet joints and cartilage matrix visualized by HE, toluidine blue, and safranin O/Fast Green staining (Scale bars, 625 µm, 200 µm). D) Volcano plot showing significantly differentially expressed proteins revealed by the 4D label‐free proteomic analysis (log FC < −1.5 or log FC > 1.5, *p* < 0.05). Red and blue dots represent proteins with increased and decreased expression in FJOA, respectively. E) Heat map showing the distribution of significantly differentially expressed proteins. Red and blue colors indicate high and low expression, respectively (*n* = 8). F) Proteomic‐based Kyoto Encyclopedia of Genes and Genomes (KEGG) analysis of the major biological pathways enriched in FJOA. G) KEGG enrichment analysis based on proteins with increased expression in FJOA. H) mRNA expression levels of genes encoding focal adhesion‐related proteins *THBS4*, *TLN2*, and *CHAD* mRNAs following the stimulation with 10 ng mL^−1^ IL‐1β for 48 h. Values were normalized by β‐actin mRNA levels (*n* = 3). I) Western blot analysis of the inflammation‐induced Talin2 expression (*n* = 3). J) Cell activity after the upregulation or downregulation of Talin2 in 24–120 h measured by the CCK‐8 assay (*n* = 3). K) Western blot analysis of changes in the expression of extracellular matrix (ECM) synthases (collagen II, aggrecan, SOX9) and catabolic enzymes (ADAMTS4, ADAMTS5, MMP3, MMP13) after the upregulation or downregulation of Talin2. L) Changes in ECM synthase and catabolic enzyme mRNA expression levels after the upregulation or downregulation of Talin2 normalized by β‐actin mRNA levels (*n* = 3). M) Left, SA‐β‐gal staining of human chondrocytes after the upregulation or downregulation of Talin2 (Scale bars, 100 µm). Right, mean SA‐β‐gal staining intensity (*n* = 3). N) Top, Alcian blue staining of human chondrocytes after the upregulation or downregulation of Talin2. Bottom, mean grey value (*n* = 3). Data are presented as the mean ± S.D. *p < 0.05; **p < 0.01; ***p < 0.001.

The significance of FJOA in spinal degeneration is frequently overlooked. Radiological evaluations (computed tomography [CT] and magnetic resonance imaging) reveal hallmark features of FJOA, including facet joint space narrowing, articular surface irregularities, and periarticular soft tissue edema with inflammatory infiltration (**Figure** **1**A). Macroscopic observations demonstrated that FJOA specimens exhibited advanced degenerative changes, including full‐thickness cartilage erosion with subchondral bone plate exposure (Figure 1B). Extensive cartilage erosion and proteoglycan depletion were further confirmed by HE, toluidine blue, and safranin O/fast green staining (Figure 1C). Therefore, the discarded surgical specimens were collected for 4D label‐free proteomic analysis to explore FJOA mechanisms. Our analysis revealed 24 upregulated and 34 downregulated proteins (log fold change [FC] < −1.5 or log FC > 1.5, *p* < 0.05; Figure 1D,E). Based on the differentially expressed proteins, Kyoto Encyclopedia of Genes and Genomes (KEGG) pathway analysis was performed to identify metabolic and signal transduction pathways potentially involved in FJOA (Figure 1F). Among the upregulated proteins, the focal adhesion pathway (map04510), known to be critical for cartilage homeostasis, was significantly enriched (Figure 1G). Interleukin‐1β (IL‐1β) was applied to simulate chondrocyte inflammation to screen for proteins critical for focal adhesion (*THBS4*, *TLN2*, and *CHAD*). *TLN2* mRNA expression decreased (Figure 1H), whereas Talin2 protein expression increased (Figure 1I) following IL‐1β stimulation, warranting further investigation.

To investigate the biological function of Talin2 in FJOA, specific small interfering RNA (siRNA) and lentiviruses were constructed for targeted *TLN2* knockdown and overexpression, respectively (Figure S1A–D, Supporting Information). Talin2 overexpression significantly suppressed cellular vitality, whereas knockdown conversely enhanced it (Figure 1J). Western blotting and qRT‐PCR demonstrated that Talin2 overexpression significantly promoted the expression of ADAMT4, ADAMTS5, MMP3, and MMP13, but inhibited the expression of collagen II, aggrecan, and SOX9 (Figure 1K,L). Overexpression of Talin2‐induced chondrocyte senescence detected by senescence‐associated β‐galactosidase (SA‐β‐gal) staining, whereas Talin2 knockdown had an opposite effect (Figure 1M). Alcian staining of chondrocytes demonstrated that Talin2 overexpression impaired ECM synthesis, with Talin2 deficiency conversely promoting ECM synthesis (Figure 1N).

Collectively, these results indicate that Talin2 is a pathogenic mediator in FJOA, exhibiting elevated expression patterns and functionally driving cartilage degeneration by disrupting ECM homeostasis.

### 2.2 OTUB1 Maintains Talin2 Stability and Affects Cartilage Activity

**Figure 2:**
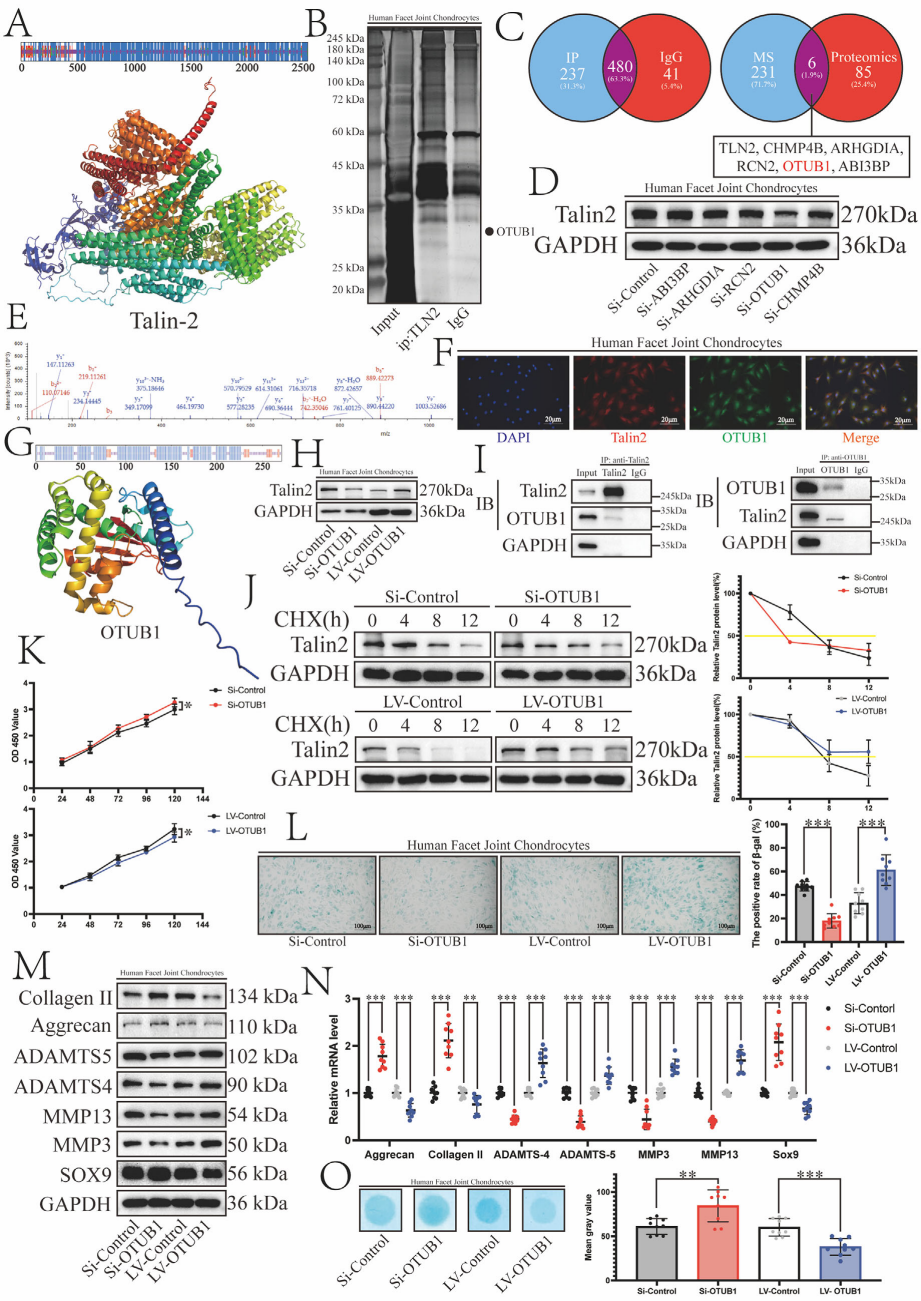
Ovarian tumor domain‐containing ubiquitin aldehyde‐binding protein 1 (OTUB1) maintains Talin2 stability and affects cartilage activity. A) Top, Talin2 secondary structure predicted by PRABI. Red represents extended strand (5.59%), blue represents α helix (67.74%), yellow represents random coil (23.96%), and green represents β turn (2.71%). Bottom, Talin2 spatial structure predicted by AlphaFold3. B) Silver staining of the immunoprecipitated Talin2‐associated proteins. The samples were qualitatively analyzed by mass spectrometry. C) Immunoprecipitation of Talin2‐associated proteins (237, 31.3%) identified from the intersection with proteomic data. D) Western blot detection of changes in Talin2 expression after knockdown of candidate proteins. E) OTUB1 mass spectrometry analysis. F) Polychromatic immunofluorescence staining revealing spatial co‐localization of Talin2 and OTUB1. G) Top, OTUB1 secondary structure predicted by PRABI. Red represents extended strand (8.86%), blue represents α helix (54.98%), yellow represents random coil (32.1%), and green represents β turn (4.06%). Bottom, Talin2 spatial structure predicted by AlphaFold3. H) Expression of Talin2 after the upregulation or downregulation of OTUB1. I) Interaction between exogenous Talin2 and OTUB1 in chondrocytes investigated by co‐immunoprecipitation. J) Left, Talin2 expression in chondrocytes with OTUB1 knockdown treated with 100 µg mL^−1^ cycloheximide (CHX) for 0–12 h. Right, mean relative Talin2 protein levels normalized by GAPDH levels (*n* = 3). K) Cell activity after the upregulation or downregulation of OTUB1 in 24–120 h measured by the CCK‐8 assay (*n* = 3). L) Left, SA‐β‐gal staining of human chondrocytes after the upregulation or downregulation of OTUB1 (Scale bars, 100 µm). Right, mean SA‐β‐gal staining intensity (*n* = 3). M) Western blot analysis of changes in the expression of ECM synthases (collagen II, aggrecan, SOX9) and catabolic enzymes (ADAMTS4, ADAMTS5, MMP3, MMP13) after the upregulation or downregulation of OTUB1. N) Changes in ECM synthase and catabolic enzyme mRNA expression levels after the upregulation or downregulation of OTUB1 normalized by β‐actin mRNA levels (*n* = 3). O) Left, Alcian blue staining of human chondrocytes after the upregulation or downregulation of OTUB1. Right, mean grey value (*n* = 3). Data are presented as the mean ± S.D. *p < 0.05; **p < 0.01; ***p < 0.001.

Structural characterization through secondary and spatial structures identified Talin2 as an α‐helix‐enriched molecule with high conformational stability (**Figure** **2**A).^[^ ^19^ ^]^ In FJOA, qRT‐PCR revealed reduced mRNA levels of Talin2, while proteomic profiling paradoxically demonstrated elevated protein expression. This discrepancy between transcriptional suppression and protein accumulation prompted us to hypothesize the existence of regulatory binding partners that inhibit the degradation of Talin2, thereby stabilizing its expression despite diminished mRNA availability. Immunoprecipitation‐mass spectrometry analysis, combined with proteomics, was performed to identify proteins that interact with Talin2 and modulate its expression (unique peptides > 2; log FC < −1.2 or log FC > 1.2, *p* < 0.05; Figure 2B,C). The expression of the candidate proteins was inhibited to verify their association with Talin2 (Figure 2D). The targeted knockdown of OTUB1 resulted in a concomitant reduction in Talin2 expression, suggesting a regulatory relationship between these two proteins (Figure S1E, Supporting Information). Figure 2E presents the mass spectrometry analysis confirming the identity of OTUB1. Polychromatic immunofluorescence staining showed that Talin2 and OTUB1 co‐localized in the cytoplasm of chondrocytes (Figure 2F). As illustrated in Figure 2G, the predicted secondary and spatial structures of OTUB1 show a canonical α/β‐fold architecture accompanied by a conformationally dynamic N‐terminal domain. When OTUB1 in chondrocytes was knocked down or overexpressed artificially, the expression of Talin2 also decreased or increased, respectively (Figure 2H; Figure S1F, Supporting Information). In contrast, knockdown or overexpression of Talin2 did not affect OTUB1 expression (Figure S1G, Supporting Information). Subsequent investigations focused on elucidating the regulatory mechanism by which OTUB1 modulates Talin2 expression. Endogenous co‐immunoprecipitation (Co‐IP) assays confirmed the interaction between OTUB1 and Talin2 under intracellular conditions (Figure 2I). Validation through cycloheximide (CHX) experiments demonstrated that OTUB1 overexpression significantly extended Talin2 half‐life, whereas OTUB1 knockdown accelerated Talin2 degradation (Figure 2J). These findings collectively establish OTUB1 as a critical stabilizer of Talin2 through interaction.

In terms of functionality, OTUB1 overexpression significantly suppressed cellular metabolic activity and potentiated senescence, whereas OTUB1 knockdown enhanced proliferative capacity and attenuated senescence (Figure 2K,L). At the molecular level, OTUB1 overexpression upregulated catabolic enzymes, while concurrently downregulating matrix synthesis enzymes. Conversely, OTUB1 knockdown reciprocally enhanced matrix synthesis enzymes and suppressed catabolic enzymes (Figure 2M,N). This OTUB1‐mediated dysregulation of ECM equilibrium was further confirmed by the Alcian blue experiment (Figure 2O).

These findings collectively demonstrate that OTUB1 stabilizes Talin2 through interaction while driving ECM dysregulation via catabolic and anabolic pathways.

### 2.3 OTUB1 Interacts with Talin2 and Promotes Its K48‐Linked Non‐Canonical Deubiquitination

**Figure 3:**
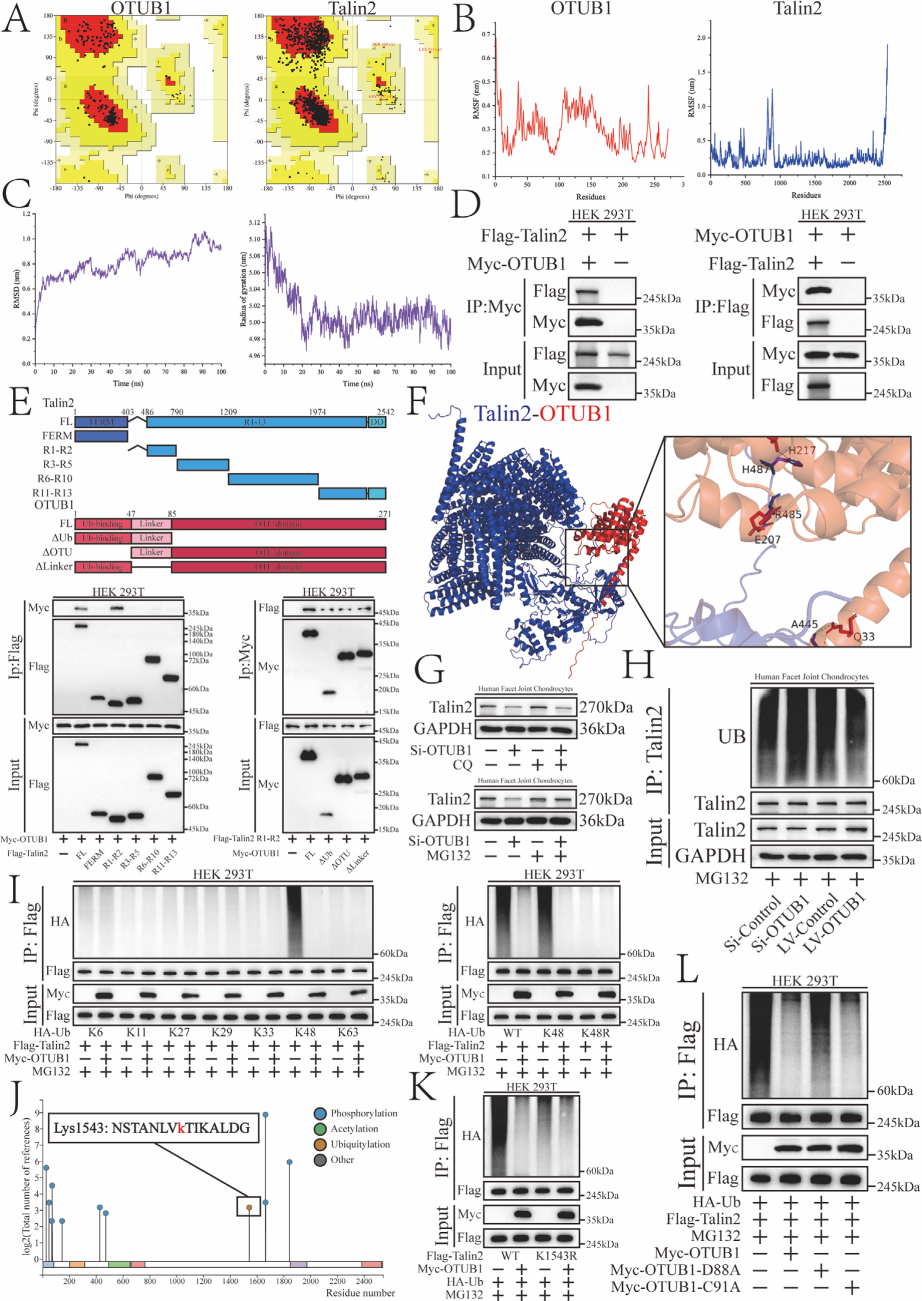
OTUB1 interacts with Talin2 and promotes its K48‐linked non‐canonical deubiquitination. A) Ramachandran plots of Talin2 and OTUB1 visualizing dihedral angles *ψ* and *φ* of the amino acid residues, which are divided into the most favored regions (red, Talin2 = 95.4%, OTUB1 = 94.3%), additional/generously allowed regions (yellow, Talin2 = 4.5%, OTUB1 = 5.7%), and forbidden regions (white, Talin2 = 0%, OTUB1 = 0%). B) RMSF graph showing flexible regions of Talin2 and OTUB1. C) RMSD and Rg graphs showing the Talin2/OTUB1 interaction stability. D) Co‐immunoprecipitation of exogenous Talin2 and OTUB1 in HEK293T cells. E) Top, schematic diagram illustrating the truncated mutants of Talin2 and OTUB1. Bottom, determination of the Talin2/OTUB1 interaction region by co‐immunoprecipitation. F) Left, the spatial structure of the Talin2/OTUB1 complex. Blue and red colors represent Talin2 and OTUB1, respectively. Right, predictive binding sites of the Talin2/OTUB1 interaction. G) Western blot analysis of Talin2 expression in chondrocytes with OTUB1 knockdown treated with 20 µm CQ or 10 µm MG132 for 6 h. H) Western blot analysis of Talin2 ubiquitination in chondrocytes after the upregulation or downregulation of OTUB1. I) Left, ubiquitination sites detected through the indicated types of ubiquitin (K6, K11, K27, K29, K33, K48, and K63, with the intact Lys residue alone). Right, a ubiquitination site detected through the indicated type of ubiquitin (K48R, only residue Lys48 was mutated). J) Talin2 ubiquitination sites predicted by PhosphoSitePlus, suggesting that Lys1543 is a potential site. K) Mutation of lysine 1543 of Talin2 to arginine for ubiquitination detection. L) Mutation of OTUB1 aspartic acid 88 and cystine 91 to alanine validating the deubiquitination mechanism of OTUB1 action.

Our subsequent investigations will focus on elucidating the molecular mechanism of the Talin2/OTUB1 interaction, specifically characterizing the form and dynamics of their interaction through structural biology approaches. Ramachandran plots were used to assess the rationality of the forecasted structure visualizing dihedral angles *ψ* and *φ* of the amino acid residues (**Figure** **3**A). The structures were all reasonably consistent with the rules of stereochemistry, without amino acid residues located in forbidden regions. The structures of OTUB1 and Talin2 were docked using HDOCK (Docking Score = −233.51, confidence score = 0.8416),^[^ ^20^ ^]^ which was further simulated using molecular dynamics (MD, Figure S1H, Video S1, Supporting Information).^[^ ^21^ ^]^ The root mean square fluctuation (RMSF) represents the fluctuation and flexibility of the residues. Flexible regions were identified in the middle and tail of Talin2, which adapted to OTUB1 through self‐configuration (Figure 3B). The root mean square deviation (RMSD) and radius of gyration (Rg) calculations indicated that the OTUB1/Talin2 complex was stable in MD (Figure 3C). Exogenous coimmunoprecipitation (Co‐IP) was consistent with MD simulations and indicated a direct interaction between OTUB1 and Talin2 (Figure 3D). Molecular mapping assays revealed residues 404–790 of Talin2 responsible for binding to OTUB1 (Figure 3E). Furthermore, in the Talin2/OTUB1 complex, amino acids A445, R485, and H487 of Talin2 were predicted to interact with amino acids Q33, E207, and H217 of OTUB1, respectively (Figure 3F).

Given the classification of OTUB1 as a deubiquitinase, we further investigated whether deubiquitination is involved in its interaction with Talin2. The downregulation of Talin2 induced by OTUB1 knockdown was reversed by MG132 rather than by chloroquine, suggesting that OTUB1 regulates Talin2 stability in a proteasome‐mediated manner (Figure 3G). The ubiquitination of Talin2 was affected by the artificial overexpression and knockdown of OTUB1 (Figure 3H). OTUB1 interfered with the K48‐linked ubiquitination of Talin2 (Figure 3I). The ubiquitination site of Talin2 was predicted using PhosphoSitePlus and verified by constructing mutants (Figure 3J,K; Figure S1I, Supporting Information).^[^ ^22^ ^]^ The inconsistency between the ubiquitination site of Talin2 and OTUB1 binding region suggests that the effect of OTUB1 did not depend on its deubiquitinating enzyme activity. The assay showed that OTUB1^C91A^ (disrupts deubiquitination enzyme activity) effectively promoted deubiquitination, whereas OTUB1^D88A^ (disrupts interaction with E2‐binding enzymes) was ineffective (Figure 3L).

In summary, OTUB1 directly interacts with Talin2 and mediates its stabilization through a non‐canonical deubiquitination mechanism.

### 2.4 Talin2 Selectively Regulates the Expression of *CCL2* in Chondrocytes

**Figure 4:**
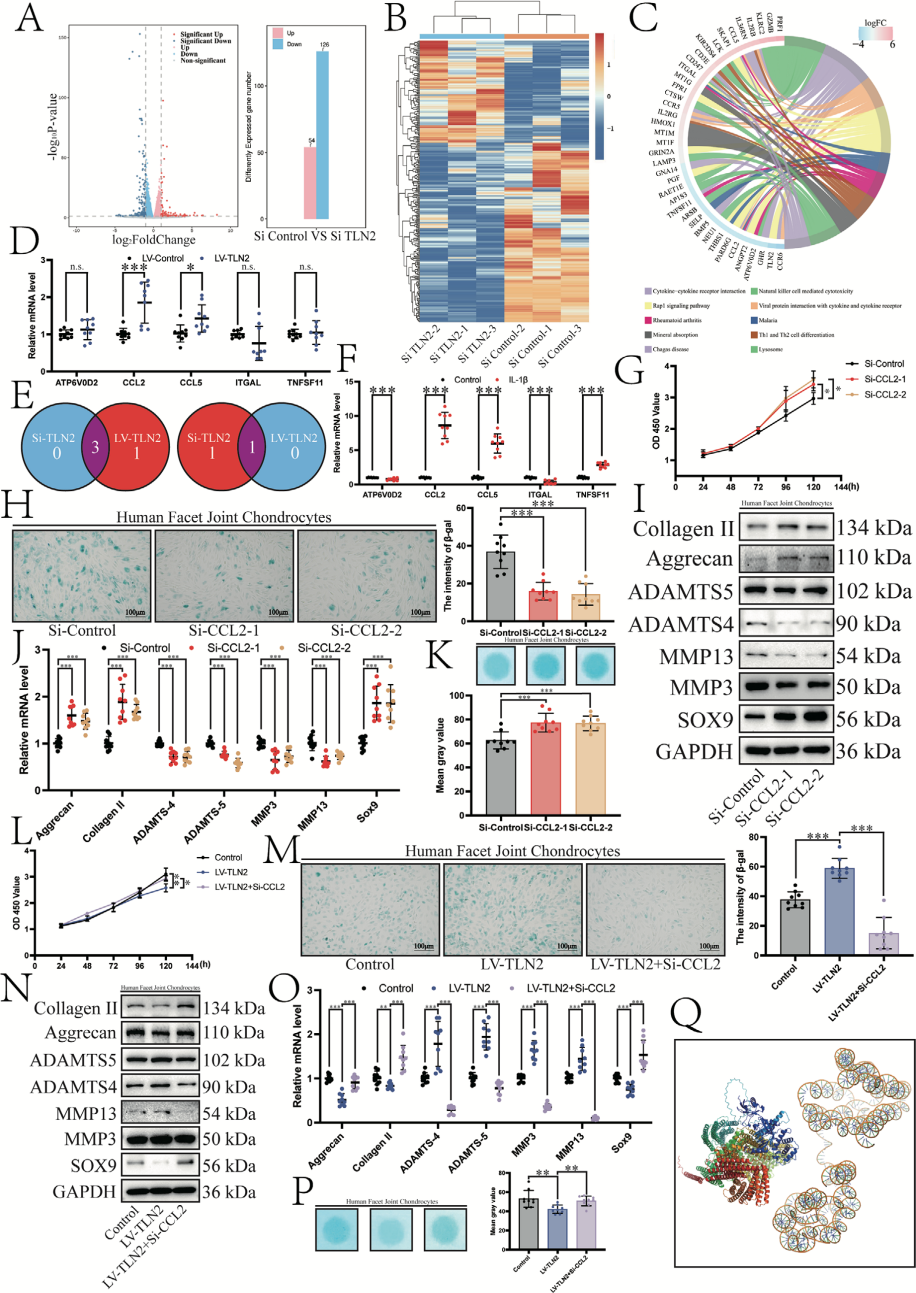
Talin2 selectively regulates *CCL2* expression in chondrocytes. A) Left, volcano plot showing significantly differentially expressed genes (DEGs) after Talin2 downregulation (log FC < −2 or log FC > 2, *p* < 0.05). Red and blue dots represent genes with increased and decreased expression, respectively. Right, bar chart of significant DEGs. B) Heat map illustrating the distribution of significant DEGs. Red and blue colors represent genes with increased and decreased expression, respectively (*n* = 6). C) KEGG enrichment analysis of the major biological pathways represented in DEGs based on RNA sequencing. D) Expression levels of rheumatoid arthritis‐related genes after Talin2 upregulation. Values were normalized by β‐actin mRNA levels (*n *= 3). E) Screening of genes affected by changes in Talin2 expression. F) Expression levels of rheumatoid arthritis‐related genes after the stimulation with 10 ng mL^−1^ IL‐1β for 48 h. Values were normalized by β‐actin mRNA levels (*n* = 3). G) Cell activity was evaluated after the downregulation of *CCL2* in 24–120 h by the CCK‐8 assay (*n* = 3). H) Left, SA‐β‐gal staining of human chondrocytes after the downregulation of *CCL2* (Scale bars, 100 µm). Right, mean SA‐β‐gal staining intensity (*n* = 3). I) Changes in protein levels of ECM synthases and catabolic enzymes after the downregulation of *CCL2*. J) Changes in mRNA expression levels of ECM synthases and catabolic enzymes after the downregulation of *CCL2*. Values were normalized by β‐actin mRNA levels (*n* = 3). K) Top, Alcian blue staining of human chondrocytes after the downregulation of *CCL2*. Bottom, mean greyscale values (*n* = 3). L) Cell activity after the upregulation of Talin2 with or without the downregulation of *CCL2* in 24–120 h by CCK‐8 (*n* = 3). M) Left, SA‐β‐gal staining of human chondrocytes after upregulation of Talin2 with or without the downregulation of *CCL2* (Scale bars, 100 µm). Right, mean SA‐β‐gal staining intensity (*n* = 3). N) Changes in protein expression of ECM synthases and catabolic enzymes after the upregulation of Talin2 expression with or without the downregulation of *CCL2* expression. O) Changes in mRNA expression levels of ECM synthases and catabolic enzymes after the upregulation of Talin2 with or without the downregulation of *CCL2*. Values were normalized by β‐actin mRNA levels (*n* = 3). P) Left, Alcian blue staining of human chondrocytes after the upregulation of Talin2 with or without the downregulation of *CCL2*. Right, mean greyscale values (*n* = 3). Q) Predicted interaction of Talin2 and *CCL2*. Data are presented as the mean ± S.D. *p < 0.05; **p < 0.01; ***p < 0.001.

To elucidate the pathophysiological role of Talin2 in FJOA development, we will conduct a comprehensive investigation of its molecular mechanisms within chondrocyte biology. When Talin2 expression was inhibited, chondrocyte samples were analyzed by transcriptome RNA sequencing to identify downstream target genes. A total of 54 upregulated and 126 downregulated genes were filtered (log FC < −2 or log FC > 2, *p* < 0.05; **Figure** **4**A,B). KEGG pathway analysis of differentially expressed genes revealed the most enriched pathways, with genes involved in rheumatoid arthritis being significantly associated to FJOA (Figure 4C). To validate candidate targets identified in RNA sequencing, transcript levels of genes were systematically re‐examined following Talin2 overexpression in chondrocytes (Figure 4D). Knockdown or overexpression of Talin2 downregulated or upregulated *CCL2*, respectively. Furthermore, IL‐1β markedly elevated *CCL2* expression in chondrocytes, revealing a coordinated regulatory mechanism between Talin2 and *CCL2* in cartilage pathophysiology (Figure 4E,F).

Therefore, *CCL2* was presumed to be a downstream gene of Talin2, that was specifically knocked down to further explore its biological functions (Figure S1J, Supporting Information). When the expression of *CCL2* was inhibited, the activity of chondrocytes was promoted and senescence progression was inhibited (Figure 4G,H). Knockdown of *CCL2* in chondrocytes induced a catabolic–anabolic imbalance characterized by upregulated expression of matrix‐synthesizing enzymes and concomitant suppression of matrix‐degrading proteases (Figure 4I,J). This metabolic reprogramming resulted in the accumulation of ECM components, as quantified by Alcian blue analysis (Figure 4K).

To mechanistically validate *CCL2* as a downstream effector of Talin2 in chondrocytes, we conducted rescue experiments (Figure S1K, Supporting Information). In Talin2‐overexpressing chondrocytes, established via lentiviral transduction, subsequent *CCL2* knockdown significantly attenuated the Talin2‐induced phenotypic alterations. Notably, *CCL2* depletion rescued both the Talin2‐mediated suppression of cell proliferation and acceleration of senescence (Figure 4L,M). Concomitantly, *CCL2* knockdown reversed the Talin2‐driven metabolic imbalance by normalizing matrix‐degrading protease overexpression while restoring matrix‐synthesizing enzymes (Figure 4N,O). Alcian blue quantification confirmed that *CCL2* ablation effectively mitigated Talin2‐induced ECM catabolism (Figure 4P). AlphaFold3 did not predict Talin2 and *CCL2* interactions, indicating that the mechanism of the relationship observed by us needs to be further explored (Figure 4O).

These data suggest that *CCL2* is a functional downstream target of Talin2 that critically modulates chondrocyte homeostasis. The Talin2/*CCL2* axis governs cartilage matrix metabolism, suggesting therapeutic potential for targeting this pathway in FJOA.

### 2.5 Talin2 Knockdown Inhibits FJOA In Vivo

**Figure 5:**
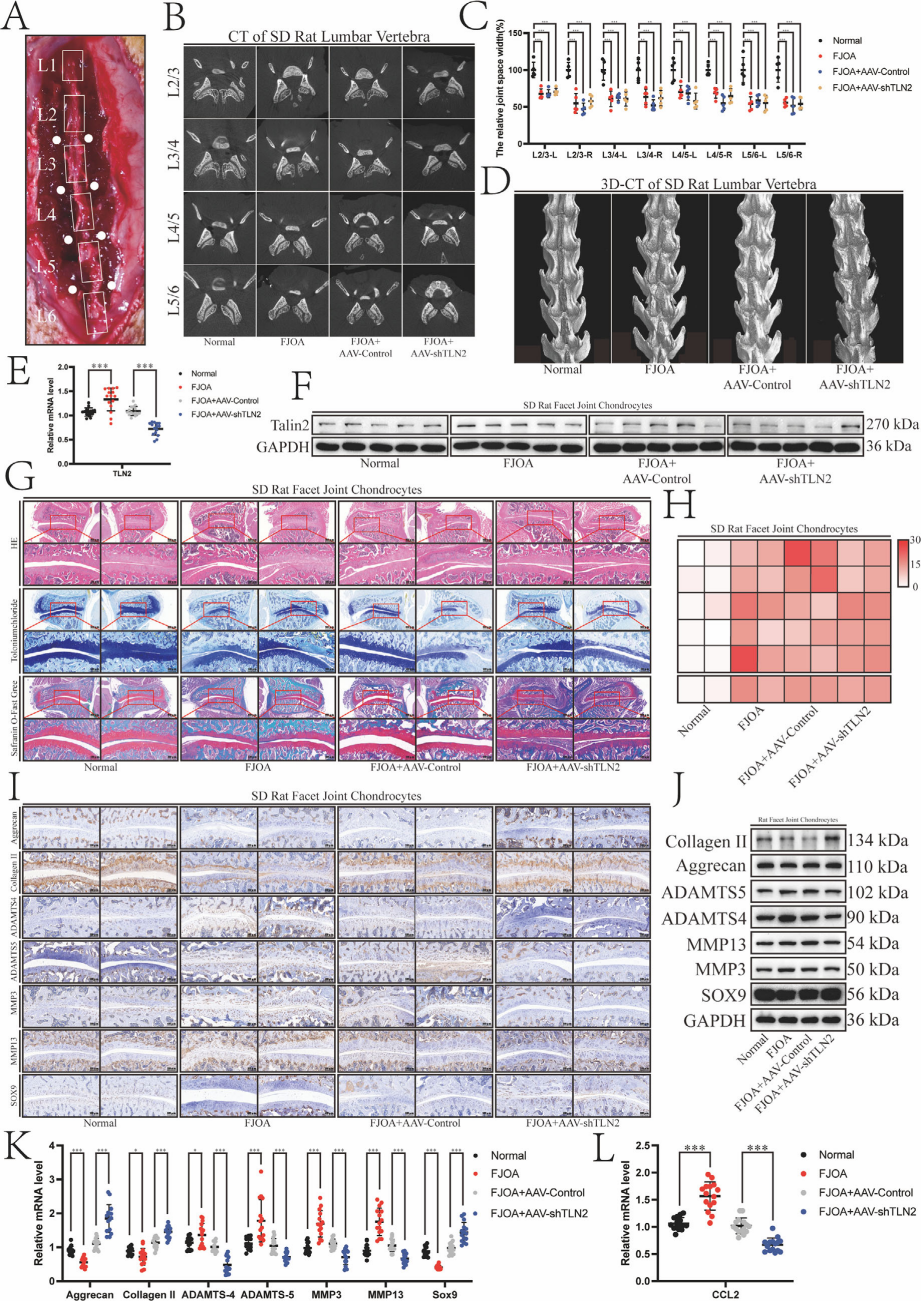
Talin2 knockdown inhibits FJOA in vivo. A) Posterior midline incision revealing bilateral facet joints located according to spinal spinous processes (L1‐6) in an SD rat in the prone position. White dots represent the position of facet joints (L2/3, L3/4, L4/5, L5/6). B) Transverse CT scan of lumbar intervertebral structures. C) Relative bilateral facet joint space width measurements. D) 3D‐CT of the lumbar spine of an SD rat. E) *TLN2* mRNA expression levels in L4/5 facet joints of SD rats determined by qRT‐PCR. Values were normalized by β‐actin mRNA levels (*n* = 20). F) Talin2 protein levels in L5/6 facet joints of SD rats determined by western blotting. G) L3/4 facet joints and cartilage matrix of SD rats stained by HE, toluidine blue, and safranin O/Fast Green (Scale bars, 500 µm, 100 µm). H) Top, heat map of OARSI scores of the facet joints and cartilage matrix of SD rats. Bottom, mean OARSI score of each group. I) ECM synthases and catabolic enzymes in the L2/3 cartilage matrix of SD rat visualized by immunohistochemistry staining (Scale bars, 100 µm). J) Protein levels of ECM synthases and catabolic enzymes in SD rats (L5/6) determined using western blotting. K) mRNA expression levels of ECM synthases and catabolic enzymes in SD rats. Values were normalized by β‐actin mRNA levels (L4/5, *n* = 20). L) *CCL2* mRNA expression level in SD rats determined by qRT‐PCR. Values were normalized by β‐actin levels (L4/5, *n* = 20). Data are presented as the mean ± S.D. *p < 0.05; **p < 0.01; ***p < 0.001.

To validate the Talin2/*CCL2* axis in vivo, we established an FJOA animal model. The bilateral facet joints of SD rats were positioned based on the spinal spinous processes. The capsules of which (L2/3, L3/4, L4/5, L5/6) were gently punctured using a microinjector to induce FJOA (**Figure** **5**A; Figure S1L, Supporting Information).^[^ ^23^ ^]^ Sham surgery was performed in the control group, and adeno‐associated viruses (AAV‐control or AAV‐shTLN2) were injected into the experimental group to investigate the Talin2 role in vivo. In the transverse CT images, slight destruction of the lateral edge of the facet joints and narrowing of the facet joint height indicated successful FJOA modeling (Figure 5B,C). 3D‐CT directly visualized osteophyte hyperplasia of the bilateral facet joints (Figure 5D). Both qRT‐PCR and Western blotting results confirmed specific downregulation of Talin2 expression (Figure 5E,F; Figure S1M, Supporting Information). Bilateral facet joints from the L3/4 spinal segments were harvested for histological analysis. HE staining revealed joint architecture alterations consistent with FJOA. Toluidine blue and safranin O/fast green staining demonstrated articular cartilage degeneration, characterized by matrix loss and proteoglycan depletion (Figure 5G). Histopathological features were quantitatively analyzed using the OARSI score (Figure 5H).^[^ ^24^ ^]^ Despite not mitigating macroscopic FJOA progression, Talin2 knockdown demonstrated significant chondroprotective effects at the molecular level. Multimodal analyses (immunohistochemistry, Western blotting, and qRT‐PCR) revealed Talin2 suppression enhanced the expression of ECM anabolic enzymes while downregulating catabolic mediators in chondrocytes (Figure 5I–K), suggesting a potential mechanism for extracellular matrix homeostasis maintenance. Furthermore, Talin2 knockdown suppressed *CCL2* expression (Figure 5L), functionally establishing *CCL2* as a downstream effector of Talin2.

These in vivo investigations conclusively demonstrate the central role of Talin2 in regulating the ECM metabolic equilibrium during FJOA pathogenesis. This animal model validation establishes the Talin2/*CCL2* axis as a regulatory mechanism governing ECM homeostasis in FJOA.

### 2.6 Mechanical Load Induces Talin2 Expression

**Figure 6:**
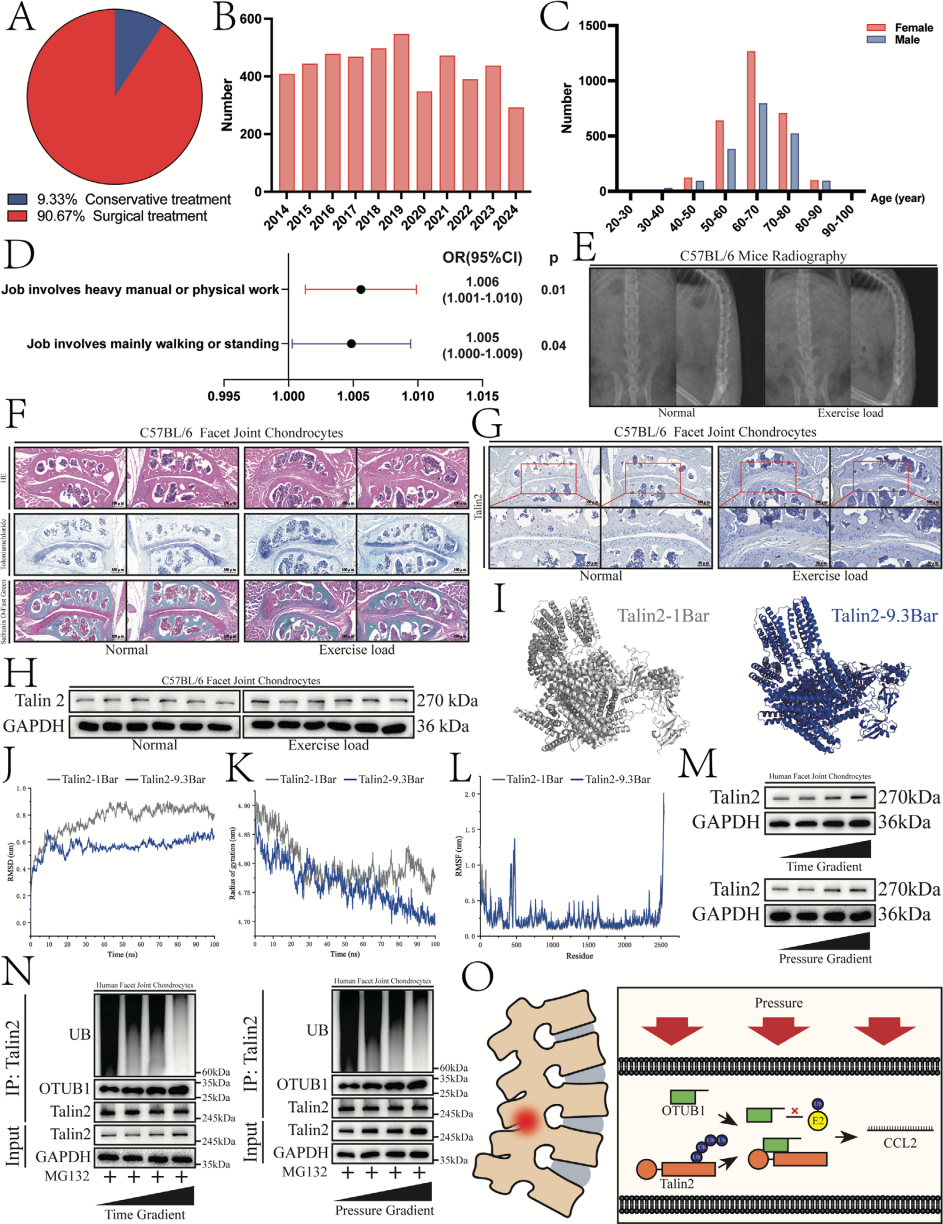
Mechanical load augments Talin2 expression. A) Pie chart showing fractions of patients in the Peking Union Medical College Hospital that underwent conservative (9.33%) and surgical (90.67%) treatment of FJOA in 2014–2024. B) Bar graph illustrating the fluctuation in the number of patients with FJOA in 2014–2024. C) Bar graph showing sex‐ and age‐dependent distributions of patients with FJOA. D) Associations of genetically predicted workload (ukb‐b‐2002, ukb‐b‐4461) with lumbar intervertebral joint operation (ukb‐b‐19054). CI, confidence interval; OR, odds ratio. E) X‐ray images of the frontal and lateral lumbar spines of C57BL/6 mice in normal and exercise load groups. F) Facet joint and cartilage matrix sections of the normal and exercise load groups stained by HE, toluidine blue, and safranin O/Fast Green (Scale bars, 100 µm). G) Talin2 expression in the cartilage matrix of the normal and exercise load groups visualized by immunohistochemistry staining (Scale bars, 100 µm, 50 µm). H) Talin2 protein expression in the cartilage matrix of the normal and exercise load groups. I) Spatial structure of Talin2 under 1 bar (grey) and 9.3 bar (blue) of pressure. J) RMSD calculations showing compression of Talin2 under 9.3 bar. K) Rg measurements indicating compression of Talin2 under 9.3 bar. L) RMSF calculations showing changes in the flexible regions of Talin2 under 9.3 bar. M) Top, time course of Talin2 protein expression in chondrocytes (0, 2, 4, and 8 h) under a constant pressure (6 bar). Bottom, changes in Talin2 protein expression in chondrocytes during varying pressure gradient (1, 3, 6, and 9 bar) for 8 h. N) Left, the time course of OTUB1 expression and ubiquitination in chondrocytes (0, 2, 4, and 8 h) under constant pressure (6 bar). Right, changes in OTUB1 expression and ubiquitination in chondrocytes during varying pressure gradient (1, 3, 6, and 9 bar) for 8 h. O) Diagram illustrating the Talin2/*CCL2* pathway and stabilization and deubiquitination of Talin2 by OTUB1.

Previous results have demonstrated that Talin2 in FJOA significantly upregulates CCL2, which exhibits pro‐inflammatory properties by mediating immune cell recruitment.  This pathological cascade amplifies the inflammatory responses, thereby exacerbating FJOA. To further delineate the molecular pathogenesis of FJOA, the upstream regulatory mechanisms responsible for Talin2 overexpression in FJOA were investigated. Therefore, this retrospective clinical study was conducted to explore the etiological factors contributing to FJOA. Patients diagnosed at Peking Union Medical College Hospital between August 2013 to August 2024 were screened based on radiological results and chief complaints. Among 4781 patients, 4335 (90.67%) underwent surgical treatment (posterior lumbar decompression or regional neural blockade), and 446 (9.33%) received conservative treatment (**Figure** **6**A). Demographic analysis demonstrated a comparable annual patient volume, with peak disease prevalence observed in individuals aged 60–70 years (43.11%). Notably, the cohort included 59.72% female and 40.28% male patients (Figure 6B,C). Furthermore, in the Mendelian randomization analysis, jobs involving heavy manual or physical work (ukb‐b‐2002, odds ratio [OR] = 1.006, *p* = 0.01) and those involving predominantly walking or standing (ukb‐b‐4461, OR = 1.005, *p* = 0.04) were positively correlated with lumbar intervertebral joint operations (ukb‐b‐19054) (Figure 6D; Figure S1N,O, Tables S3, S4, Supporting Information).^[^ ^25^ ^]^ Therefore, FJOA can be considered a degenerative spinal disease associated with mechanical loading.

While Talin2 has been mechanistically linked to integrin‐mediated mechanotransduction (Figure S1P, Supporting Information),^[^ ^26^ ^]^ facilitating cellular perception of extracellular mechanical stimuli, the precise pathophysiological correlation between Talin2 expression levels and facet joint mechanical loading remains to be experimentally elucidated. To validate this hypothesis, we subjected C57BL/6 mice to inclined treadmill training, simulating human bipedal loading patterns. Although X‐ray analysis demonstrated no significant differences between the two groups (Figure 6E), histological observations revealed synovial hyperplasia and upregulated Talin2 expression in the mechanical load group (Figure 6F,G; Figure S1Q, Supporting Information). These findings demonstrate that sustained mechanical load induces FJOA pathogenesis and promotes Talin2 overexpression, establishing mechanical overloading as a critical etiological contributor to FJOA progression (Figure 6H; Figure S1R, Supporting Information).

Our subsequent investigation will systematically elucidate the molecular mechanisms underlying the upregulation of Talin2 in response to mechanical load. Previous biomechanical studies demonstrated that facet joint contact pressure reaches 0.93 MPa under combined loading conditions of 15 N m extension moment and 700 N compressive force.^[^ ^27^ ^]^ MD simulations revealed changes in the spatial structure of Talin2 induced by mechanical load (Figure 6I). MD simulations demonstrate that mechanical loading induces structural compression of Talin2 while enhancing the conformational flexibility of its random coil (residues 404–790), which serves as the binding region of OTUB1 (Figure 6J–L). These results suggest that mechanical loading enhances the Talin2/OTUB1 binding affinity. An adjustable mechanical device was engineered to recapitulate physiologically relevant mechanical loads within in vitro cellular systems. Talin2 expression is upregulated in response to both elevated mechanical load intensity and prolonged load duration. This mechanoresponsive behavior confirms that Talin2 expression is directly regulated by external mechanical stimuli (Figure 6M). Experimental analyses reveal that mechanical loading potentiates the specific binding affinity of OTUB1 to Talin2 in a load‐dependent manner, along with enhanced deubiquitination activity. These findings align with MD simulations, which demonstrate that mechanical load stabilizes Talin2 expression in chondrocytes by both promoting OTUB1 binding and enhancing deubiquitination (Figure 6N).

In summary, mechanical load, a pathomechanical risk factor for FJOA, induced the upregulation of Talin2 expression, which is subsequently stabilized via OTUB1‐mediated deubiquitination and molecular complex formation (Figure 6O).

## 3 Discussion

In this study, we demonstrate that Talin2, a large focal adhesion protein overexpressed in FJOA, regulates ECM catabolic and anabolic processes. Its interaction with OTUB1 stabilizes intracellular Talin2 levels through a non‐canonical deubiquitination mechanism. Mechanistic investigations revealed that Talin2 modulates *CCL2*, which recruits immunocytes and promotes their migration to infiltrate inflammatory sites.^[^ ^28^ ^]^ Crucially, mechanical loading induces conformational remodeling of Talin2, enhancing its binding affinity for OTUB1 and promoting its overexpression in FJOA. This mechanosensitive Talin2/OTUB1 axis establishes a feedforward loop that converts biomechanical stimuli into sustained pro‐inflammatory signaling, linking mechanical stress to FJOA progression. Beyond elucidating the pathomechanism underlying mechanical load‐induced FJOA, these results identify the Talin2/OTUB1/*CCL2* interaction, offering strategies for both prophylactic and therapeutic interventions in FJOA.

Epidemiological data from the Framingham Heart Study reveal the age‐dependent progression of FJOA, with prevalence rising to 89.2% in individuals aged 60–69 years, primarily at the L4–L5 spinal level.^[^ ^1^ ^]^ Although FJOA is now recognized as a key driver of spinal degeneration, current therapeutic strategies remain confined to palliative pain management,^[^ ^29^ ^]^ underscoring the urgent need to elucidate its pathogenesis and develop disease‐modifying interventions.^[^ ^30^ ^]^ Biomechanically, facet joints play two roles: distributing axial loads and stabilizing spinal motion during flexion/rotation.^[^ ^4^ ^]^ When the spine was subjected to a 700 N compressive axial load (25 N s^−1^), followed by a 15 Nm extension moment (0.25 Nm s^−1^), the mean pressure in the facet joint was 0.93 MPa, with a peak pressure of 3.73 MPa.^[^ ^27^ ^]^ Our treadmill experiment in C57Bl/6J mice confirmed the hypothesis that mechanical overloading is a high‐risk factor for FJOA.^[^ ^31^ ^]^

Facet joint cartilage is composed of collagen fiber arrangements, glycosaminoglycans, and proteoglycans that exhibit zonal structural adaptations to withstand compressive, tensile, and shear forces.^[^ ^32^ ^]^ In contrast, the articular marginal cartilage matrix, protected by synovial folds and meniscoids, is thinner, sensitive to mechanical loads, and susceptible to trauma.^[^ ^33^ ^]^ The difference in cartilage coverage is thought to explain the higher prevalence of FJOA in females.^[^ ^34^ ^]^ Chondrocytes dynamically regulate matrix homeostasis through catabolic/anabolic balancing; however, mechanical overstress disrupts this equilibrium, triggering matrix degradation, subchondral bone remodeling, synovitis, and osteophytogenesis.^[^ ^35^ ^]^ These structural changes propagate neuroinflammatory cascades via nerve branch activation (manifesting as facet syndrome),^[^ ^36^ ^]^ while ligamentum flavum hypertrophy and osteophyte expansion exacerbate spinal canal stenosis.^[^ ^37^ ^]^ Therefore, an effective therapeutic strategy for chondrocyte repair should enhance mechanoadaptation and intercept inflammatory pathways to prevent degenerative progression.

Our study revealed pathological overexpression of Talin2 in FJOA, where it regulates the expression of *CCL2* through direct interaction with OTUB1 via deubiquitination. These findings elucidate how aberrant mechanical loading is transduced into inflammatory signaling cascades, clarifying both high‐risk biomechanical drivers and molecular pathways underlying FJOA pathogenesis.^[^ ^38^ ^]^ This mechanistic insight holds significant public health relevance, informing strategies to mitigate modifiable risk factors. Crucially, we identified a specific interaction between Talin2 and OTUB1, enabling a novel precision therapeutic strategy: targeted small‐molecule inhibitors designed to disrupt Talin2/OTUB1 binding affinity.^[^ ^39^ ^]^ This approach achieves dual therapeutic effects by accelerating Talin2 degradation, blocking mechano‐inflammatory signal conversion, and promoting ECM synthesis. This targeted strategy not only alleviates chronic low back pain but alters disease trajectory by preserving facet joint structural integrity and decelerating spinal degeneration, addressing critical unmet needs in aging populations through synergistic symptom‐structure management while reducing surgical burden and advancing cost‐effective precision therapeutics for musculoskeletal aging.

This study acknowledges methodological constraints that warrant consideration. First, the single‐center retrospective design introduces selection bias, potentially masking geographic, lifestyle, and ethnic determinants of FJOA susceptibility. Future multi‐center collaborative studies with stratified demographic sampling are required to strengthen evidence‐based validity. Second, while AlphaFold3‐enabled structural predictions of Talin2 and OTUB1 proteins provided cost‐effective insights for molecular docking and MD simulations, overcoming traditional structural biology constraints, the inherent limitations of AlphaFold3 are evident.^[^ ^20^, ^40^ ^]^ Specifically, the inability to accurately model random coil conformations in Talin2 introduces structural ambiguity, and computational predictions alone remain insufficient to replace experimental validation of disease mechanisms.^[^ ^41^ ^]^ Achieving reliable sequence‐structure‐function correlations will require continuous large‐scale model training with experimental benchmarking.^[^ ^42^ ^]^ Furthermore, our in vitro mechanobiology device, though demonstrating load‐dependent cellular responses, lacks real‐time pressure monitoring and cannot fully recapitulate the dynamic mechanical microenvironment of synovial tissues in vivo. Affinity changes inferred from expression‐level analyses provide only indirect evidence of mechanotransduction. Future investigations should prioritize visualization of conformational transitions alongside AI model refinement, bridge computational predictions with physiological reality.

FJOA represents a growing global health burden with notable early‐onset trends. Our research revealed the involvement of the Talin2/OTUB1/*CCL2* axis in mechanical load‐inflammatory conversion. These findings suggest novel preventive and therapeutic strategies for FJOA.

## 4 Conclusion

FJOA represents a growing global health burden with notable early‐onset trends. This study identifies Talin2 as a key regulator in the pathogenesis of FJOA, highlighting its overexpression, mechanosensitivity, and interaction with OTUB1 in modulating inflammatory signaling. The findings establish a mechanistic link between biomechanical stress and FJOA progression, presenting a novel therapeutic target in the Talin2/OTUB1/CCL2 axis. These insights pave the way for precision therapeutic strategies, offering the potential for both preventive and disease‐modifying interventions in FJOA. Future studies should aim to further elucidate the broader mechanotransduction pathways and explore clinical applications of these findings to improve patient outcomes.

## 5 Experimental Section

### Human Facet Joint Cartilage

The collection of human facet joint cartilage samples was approved by the Ethics Committee of the Peking Union Medical College Hospital (Beijing, China, K6337). Facet joints requiring osteotomy for surgery were collected and classified according to the ethical guidelines. The normal group included patients with idiopathic scoliosis, whereas the degenerative group included patients with lumbar spinal stenosis.

Patient information for those diagnosed with FJOA in the Peking Union Medical College Hospital between 2013–8 and 2024–8 was collected, including age, gender, height, weight, and imaging results. The diagnosis of FJOA was based on the patient's chief complaint and imaging results.

All surgical procedures and associated risks were explained to the patients in writing, and they signed a written informed consent form. The patient information is listed in Table S1 (Supporting Information).

### Cell Culture and Cell Lines

In a sterile environment, the cartilage was separated from facet joints, shredded, and soaked in 0.2% type II collagenase (9001‐12‐1, Sigma, St Louis, MO, USA) for digestion at 37 °C for 8 h). Chondrocytes were isolated and cultured in Dulbecco's modified Eagle's medium supplemented with 10% fetal bovine serum (10091130, Thermo Fisher Scientific, Waltham, MA, USA). HEK‐293T cells were obtained from the American Type Culture Collection (CL‐0005, ATCC, Manassas, VA, USA) and cultured in Dulbecco's Modified Eagle's Medium (DMEM) supplemented with 10% fetal bovine serum. The cells were cultured in a humidified incubator (51033788, Thermo Fisher Scientific, Waltham, MA, USA) at 37 °C in an atmosphere of 95% air and 5% CO_2_.

### SiRNAs, Plasmids, and Transfection with Lentiviruses and Adeno‐Associated Viruses (AAV)

SiRNAs (targeting *TLN2*, *OTUB1*, and *CCL2*), designed and synthesized by RiboBio (Guangdong, China), were transfected into chondrocytes using Lipofectamine RNAiMax (13 778 150, Invitrogen, Carlsbad, CA, USA) at 1 µL per 10^5^ cells.

*TLN2* overexpression was achieved using the clustered, regularly interspaced short palindromic repeat‐dead Cas9 (CRISPR‐dCas9) method. A specific single‐guide RNA, designed and synthesized by GeneChem (Shanghai, China), was inserted into the U6‐sgRNA‐SV40‐MS2‐P65‐HSF1‐T2A‐Neo vector. Chondrocytes were infected with lentiviruses (MOI = 30, with HitransG P) for 24 h and cultured for 48 h prior to further analysis. For *OTUB1* overexpression, a specific cDNA sequence was inserted into the Ubi‐MCS‐3FLAG‐CBh‐gcGFP‐IRES‐puromycin vector (GeneChem). Chondrocytes were infected with lentiviruses (MOI = 25, with HitransG P) for 24 h and cultured for 48 h prior to further analysis.

The plasmids for *TLN2*, *OTUB1*, and ubiquitin overexpression were constructed using the CMV enhancer‐MCS‐3flag‐polyA‐EF1A‐zsGreen‐sv40‐puromycin, CMV enhancer‐MCS‐polyA‐EF1A‐zsGreen‐sv40‐puromycin, and CMV enhancer‐MCS‐SV40‐puromycin vectors, respectively (Genechem). Plasmids were transferred into HEK‐293T cells at 1 µg DNA per 3 µL polyethylenimine (40816ES02, Yeasen, Shanghai, China).

For the modifications of AAVs, a specific sequence was inserted into the COL2A1p‐EGFP‐mir155(MCS)‐SV40 PolyA vector (GeneChem). AAVs were injected into the facet joint cavities of SD rats (2 µL, 1 × 10^12^ vg mL^−1^).

### 4D Label‐Free Proteomics Analysis

Facet joint cartilage was qualitatively and quantitatively analyzed using 4D label‐free proteomic analysis (GeneChem). Differentially expressed proteins (log fold change [FC] < −1.5 or log FC > 1.5, *p* < 0.05) were selected for further bioinformatics analysis.

### Cell Viability Assay

Chondrocytes seeded in 96‐well plates were counted using the Cell Counting Kit‐8 (CCK‐8, 40203ES60, Yeasen, Shanghai, China) assay at an optical density of 450 nm (OD_450_) after culturing for 24, 48, 72, 96, and 120 h. In the end of the culture period, chondrocytes were incubated with 10% CCK‐8 at 37 °C for 2 h. OD_450_ values were measured using a Microplate Reader (Synergy H1, BioTek, Vermont, USA).

### Western Blotting

Total protein was extracted from chondrocytes or HEK‐293T cells using radioimmunoprecipitation assay lysis buffer (HY‐K1001, MedChemExpress, Shanghai, China), phenylmethylsulphonyl fluoride (HY‐B0496, MedChemExpress), and 4× SDS‐PAGE sample loading buffer (HY‐K1100, MedChemExpress). Proteins were separated on 10% sodium dodecyl sulfate (SDS)‐polyacrylamide gels (PG112, Epizyme Biotech, Shanghai, China) and transferred to 0.22‐µm polyvinylidene difluoride (PVDF) membranes (1 620 177, Bio‐Rad, California, USA) at 260 mA for 90 min. The PVDF membranes were blocked in 5% skimmed milk and then incubated with specific antibodies at 4 °C overnight, followed by incubation with horseradish peroxidase (HRP) AffiniPure goat anti‐rabbit lgG or goat anti‐mouse lgG at 25 °C for 2 h (34850ES60, 34851ES60, Yeasen). Protein bands were visualized with Super ECL Detection Reagent (36208ES60, Yeasen) using a ChemiDoc MP imaging system (12 003 154, Bio‐Rad). GAPDH was used as endogenous control.

The following antibodies were used: anti‐Talin2 (1:1000, GTX38970, GeneTex, California, USA), anti‐Talin2 (1:1000, ab108967, Abcam, Cambridge, UK), anti‐aggrecan (1:1000, NB600‐504, Novus Biologicals, Colorado, USA), anti‐collagen II (1:1000, 28459‐1‐AP, Proteintech, Chicago, USA), anti‐ADAMTS4 (1:1000, 11865‐1‐AP, Proteintech), anti‐ADAMTS5 (1:1000, ab182795, Abcam), anti‐MMP3 (1:1000, ER1706‐77, HUABIO, Hangzhou, Zhejiang, China), anti‐MMP13 (1:1000, ET1702‐14, HUABIO), anti‐SOX9 (1:1000, 67439‐1‐Ig, Proteintech), anti‐GAPDH (1:1000, 28459‐1‐AP, Proteintech), anti‐DYKDDDDK Tag (FLAG) (1:1000, HA722780, HUABIO), anti‐Myc (1:1000, HA601081, HUABIO), anti‐HA (1:1000, HA721750, HUABIO), anti‐OTUB1 (1:1000, ab270959, Abcam), and anti‐ubiquitin (1:1000, ET1609‐21, HUABIO).

### RNA Extraction and Quantitative Reverse Transcription Polymerase Chain Reaction (RT‐qPCR)

Chondrocytes were lysed using TRIzol reagent (15596018CN, Invitrogen, Carlsbad, CA, USA), and total RNA was extracted according to the instructions of the RNA Extraction Kit (AG21023, Accurate Biology, Changsha, Hunan, China). RNA was reverse‐transcribed using the Evo M‐MLV RT Master Mix (AG11603, Accurate Biology) to synthesize cDNA. The gene‐specific primers (Sangon Biotech, Shanghai, China) and Hieff qPCR SYBR Green Master Mix (11201ES08, Yeasen) were used to perform qRT‐PCR in an Applied Biosystems QuantStudio 7 Pro instrument (Thermo Fisher Scientific). The relative mRNA gene expression levels were calculated by the 2^−ΔΔCt^ method and normalized by the β‐actin mRNA level. The primers used are listed in Table S2 (Supporting Information).

### Mass Spectrometry

Immunoprecipitated protein samples were subjected to qualitative analysis using mass spectrometry (Genechem). Identification of proteins (unique peptides > 2) was considered reliable.

### Transcriptome RNA Sequencing

Total RNA from chondrocytes was qualitatively and quantitatively analyzed by transcriptome RNA sequencing (OE Biotech Co., Ltd., Shanghai, China) after the downregulation of *TLN2* expression. The differentially expressed genes (log FC < −2 or log FC > 2, *p* < 0.05) were screened for further bioinformatics analysis.

### Senescence β‐Galactosidase, Alcian, and Silver Staining

Chondrocyte senescence was assessed using a senescence β‐galactosidase staining kit (C0602, Beyotime, Shanghai, China). The extracellular matrix of chondrocytes was analyzed after staining with an Alcian staining kit (C0153S, Beyotime). Images were captured using an inverted microscope (Nikon, Tokyo, Japan) and analyzed by ImageJ 64‐bit, U. S. National Institutes of Health, Bethesda, MD, USA; https://imagej.net/ij/).

Protein bands were visualized using a silver staining kit (P0017S, Beyotime) and images were captured using a ChemiDoc MP imaging system (12 003 154, Bio‐Rad).

### Multiplex Immunofluorescence

Chondrocyte proteins were specifically labeled with different fluorescence markers using tyramide signal amplification, an enzymatic detection method that uses HRP to label target proteins (RC0086‐45R, RecordBio, Shanghai, China). Images were captured using an inverted fluorescence microscope (Nikon).

### Co‐Immunoprecipitation Assay

The protein extract was incubated with protein A/G‐agarose beads (HY‐K0202, MedChemExpress) that had been co‐incubated with antibody diluent overnight at 4 °C. After magnetic sorting, non‐specific binding proteins were cleared. Subsequently, proteins bound to the beads were released by heat denaturation and detected by western blotting.

### Bioinformatics Analysis

Protein secondary structure was predicted using PRABI (https://npsa‐prabi.ibcp.fr/cgi‐bin/npsa_automat.pl?page = /NPSA/npsa_hnn.html). The spatial structure was predicted using Alphafold3 (https://alphafoldserver.com/). Molecular docking was simulated using HDOCK (http://hdock.phys.hust.edu.cn) and visualized using PyMol software (https://pymol.org/2/). Ubiquitination sites were predicted using PhosphoSite Plus (https://www.phosphosite.org/).

### Molecular Dynamics Simulations

Molecular dynamics simulations were performed at constant temperature and pressure with periodic boundary conditions (Gromacs 2021.7, Amber99SB all‐atom force field, SPC model). The LINCS algorithm was applied to constrain all bonds related to hydrogen atoms with a time step of 2 fs. The Particle–Mesh Ewald method was applied to reveal electrostatic interactions. The non‐bonded interaction cutoff was set to 14 Å and updated every 50 steps. The simulation conditions were set at 298.15 K and 1 bar using the V‐rescale temperature coupling and the Parrinello–Rahman method, respectively. In the pressure simulation, the system pressure was set to 1 and 9.3 bar. The system energy was minimized using the steepest descent method to eliminate any close contact between the atoms. Subsequently, 100 ps simulations (NVT and NPT) were performed at 298.15 K. Finally, the model underwent 100 ns simulation, with the conformations saved every 50 ps. The simulation results were visualized using Gromacs, VMD, and Origin software.

### Mendelian Randomization

The dataset used for this analysis was obtained from the database of genome‐wide association studies (https://gwas.mrcieu.ac.uk). Two‐sample Mendelian randomization analysis was employed to infer the causal relationship between mechanical load (datasets ukb‐b‐4461 “Job involves mainly walking or standing” and ukb‐b‐2002 “Job involves heavy manual or physical work”) and facet joint surgery (operative procedures: secondary OPCS: Z67.5 Lumbar intervertebral joint, ukb‐b‐19054). The results of this analysis are presented in Tables S3 and S4 (Supporting Information).

### Animal Models

SD rats and C57BL/6 mice were purchased from SPF Biotechnology Co., Ltd. (Beijing, China) and maintained at Beijing Experimental Animal Research Centre Co., Ltd. (Beijing, China) in accordance with the principles and procedures of the National Institutes of Health Guide for the Care and Use of Laboratory Animals. All experiments were approved by the Animal Ethics Committee (approval No. BLARC‐LAWER‐202405006).

SD rat facet joints were exposed through a midline incision in the back after animals were anaesthetized with 1.5% isoflurane, and the erector spinae muscle was stripped, punctured, and injected with a microsyringe to simulate the FJOA (normal group: sham operation, degeneration group: physiological saline injection, Degeneration + AAV Control group: AAV Control injection, Degeneration+AAV *shTLN2* group: AAV *shTLN2* injection). The bilateral facet joints were located based on the spinous muscles, which were counted upward from the pelvis.

The mechanical load in C57BL/6 mice was achieved using a treadmill (ZS‐PT‐IV, Beijing Zhongshi Dichuang Technology Development Co., Ltd., Beijing, China). The exercise load group was subjected to running on this treadmill at a speed of 15 cm s^−1^ and an angle of 30° for 20 min day^−1^ for 30 days.

### Micro Computed Tomography (µCT)

After rats were euthanized, their spinal samples were collected, fixed in 4% paraformaldehyde (G1101, Beyotime) for 48 h, and then stored in 70% ethanol. The samples were scanned by high‐resolution µCT (SKYSCAN‐1276, Bruker, MA, USA) under the relevant parameter settings (X‐ray voltage: 40 kV; source current: 100 µA, and resolution: 4 µm). 3D images were reconstructed and analyzed.

### Histology and Immunohistochemistry

The spinal samples were fixed in 4% paraformaldehyde and decalcified in 10% ethylenediaminetetraacetic acid (E1171, Beyotime). Subsequently, the samples were dehydrated, embedded in paraffin, and sectioned at 4 µm. The sections were stained with HE, toluidine blue, and safranin O/fast green (G1003, G1032, G1053, Servicebio, Wuhan, Hubei, China), and images were captured using a NIKON‐ECLIPSE‐E100 microscope and NIKON DS‐U3 camera (Nikon, Tokyo, Japan). The Osteoarthritis Research Society International scoring to assess FJOA was performed by three qualified scorers blind to diagnoses. After deparaffinization in xylene and progressive hydration in graded ethanol solutions, sections were incubated in sodium citrate antigen retrieval solution (G1202, Servicebio) at 37 °C for 30 min. The sections were blocked with 3% hydrogen peroxide and 5% bovine serum albumin in a phosphate‐buffered solution for 30 min at 37 °C. Next, sections were incubated with specific antibodies at 4 °C overnight and HRP reagent (ZSGB‐Bio, Beijing, China). Subsequently, the sections were developed using a DAB chromogenic solution (G1212, Servicebio) and counterstained with Mayer's hematoxylin solution (G1004, Servicebio). Images were captured using a NIKON‐ECLIPSE‐E100 microscope and NIKON DS‐U3 camera (Nikon) and analyzed.

The following antibodies were used: anti‐Talin2 (1:250, GTX38970, GeneTex), anti‐Aggrecan (1:400, NB600‐504, Novus Biologicals), anti‐collagen II (1:400, 28459‐1‐AP, Proteintech), anti‐ADAMTS4 (1:400, 11865‐1‐AP, Proteintech), anti‐ADAMTS5 (1:100, ab182795, Abcam), anti‐MMP3 (1:400, ER1706‐77, HUABIO), anti‐MMP13 (1:200, ET1702‐14, HUABIO), and anti‐SOX9 (1:400, ET1611‐56, HUABIO).

### Cell Mechanical Load Assay

Chondrocytes cultured in 1 mL of medium were seeded into a 10 mL sterile syringe (Weigao, Shandong, China) that was sealed and compressed to simulate mechanical loads. Excluding the volume of the medium, different atmosphere volumes were compressed to 1 mL to simulate pressure gradients that were calculated based on the compression ratios (*P*_1_ *V*_1 _= *P*_2_ *V*_2_, 9 mL to 1 mL, 9 bar; 6 mL to 1 mL, 6 bar; 3 mL to 1 mL, 3 bar; 1 mL to 1 mL, 1 bar). Chondrocytes were cultured in the pressure gradient (1, 3, 6, or 9 bar) for 8 h. In another experiment, chondrocytes were cultured for varying periods (0, 2, 4, or 8 h) under a constant pressure of 6 bar. During this period, chondrocytes were cultured with shaking at 37 °C. After the pressure was relieved, the sealing of the syringe was rechecked.

### Statistical Analysis

Statistical analysis was performed using SPSS v28.0 (IBM, New York, USA) and GraphPad Prism 10.0 (GraphPad Software, CA, USA). Data are presented as mean ± standard deviation (S.D.). Data normality was assessed using the Kolmogorov–Smirnov test. Following confirmation of normal distribution, intergroup differences were analyzed with a two‐tailed Student's *t‐*test for parametric comparisons. Nonparametric analyses were conducted using the Mann–Whitney *U* test for independent groups and the Wilcoxon signed‐rank test for paired comparisons when normality assumptions were violated. Differences were considered significant at *p* < 0.05 (*p < 0.05, **p < 0.01, ***p < 0.001).

## Conflict of Interest

The authors declare no conflict of interest.

## Author Contributions

Y.H., H.S., and H.C. contributed equally to this work. Conceptualization was done by J.S. and Y.H. Methodology was dealt by Y.H., H.S., and X.W. Investigation was done by Y.H., J.Z., H.Z., Y.J., and H.C. Visualization was done by H.C., J.D., and X.H. Funding acquisition was done by J.S. Project administration was dealt by J.S. and W.C. Supervision was done by Y.H. and H.S. Original draft was written by J.S. and Y.H. Writing the review and editing was done by J.S. and Y.H.

## Supporting information



Supporting Information

Supplemental Video 1

## Data Availability

The data that support the findings of this study are available on request from the corresponding author. The data are not publicly available due to privacy or ethical restrictions.
