# Supplementary material for: Mechanical Load‐Induced Upregulation of Talin2 through Non‐Canonical Deubiquitination of OTUB1 Drives Facet Joint Osteoarthritis Pathogenesis
Source: Adv Sci (Weinh). 2025 Apr 25;12(25):2501046. doi: 10.1002/advs.202501046 (PMC12224972; doi:10.1002/advs.202501046)
Supplement: Supplementary file 1 — Supporting Information [file ADVS-12-2501046-s001.docx]

Supporting Information

**Mechanical load-induced upregulation of Talin2 through non-canonical deubiquitination of OTUB1 drives facet joint osteoarthritis pathogenesis**

*Yizhen Huang^1, 2 ¶^, Heng Sun^1, 2 ¶^, Haojie Chen^1, 2¶^, Xiangpeng Wang^1, 3^, Junduo Zhao^1,2^, Yang Jiao^1,2^, Hongyi Zhou^1,2^, Haoyu Cai^1,2^, Jiafeng Dai^1,2^, Xuan Huang^1,2^, Weiyun Chen^2,4*^, Jianxiong Shen^1,2*^*

**Supplementary Table 1:** Patient information

| ID | Sex | Age (year) | Height (cm) | Weight (kg) | BMI |
| --- | --- | --- | --- | --- | --- |
| 50xxxx25 | female | 16 | 164 | 54.0 | 20.08 |
| 49xxxx27 | male | 67 | 158 | 54.0 | 21.63 |
| 50xxxx03 | female | 64 | 155 | 55.0 | 22.89 |
| 48xxxx60 | male | 16 | 161 | 57.0 | 21.99 |
| 50xxxx77 | female | 48 | 164 | 90.0 | 33.46 |
| 48xxxx74 | female | 40 | 165 | 64.0 | 23.51 |
| 49xxxx68 | male | 14 | 164 | 76.5 | 28.44 |
| 50xxxx42 | female | 25 | 167 | 48.0 | 17.21 |
| 50xxxx57 | female | 17 | 165 | 56.0 | 20.57 |
| 49xxxx83 | female | 20 | 166 | 45.0 | 16.33 |
| 50xxxx97 | female | 11 | 153 | 41.0 | 17.51 |
| 50xxxx62 | male | 13 | 159 | 34.0 | 13.45 |
| 50xxxx19 | male | 67 | 173 | 90.0 | 30.07 |
| 50xxxx64 | male | 45 | 170 | 110.0 | 38.06 |
| 50xxxx09 | female | 39 | 162 | 60.0 | 22.86 |
| 50xxxx13 | male | 64 | 172 | 70.0 | 23.66 |
| 44xxxx69 | male | 61 | 174 | 83.0 | 27.41 |
| 48xxxx80 | male | 22 | 169 | 61.0 | 21.36 |
| 50xxxx71 | female | 28 | 152 | 63.0 | 27.27 |
| 48xxxx09 | female | 72 | 162 | 75.0 | 28.58 |
| 50xxxx70 | female | 67 | 162 | 75.0 | 28.58 |
| 50xxxx68 | male | 13 | 159 | 46.5 | 18.39 |
| 50xxxx16 | female | 21 | 159 | 54.0 | 21.36 |
| 50xxxx39 | female | 17 | 160 | 57.5 | 22.46 |
| 50xxxx45 | female | 13 | 150 | 56.0 | 24.89 |
| 49xxxx46 | female | 44 | 151 | 59.0 | 25.88 |
| 50xxxx39 | female | 12 | 161 | 65.0 | 25.08 |
| 51xxxx43 | female | 18 | 168 | 62.0 | 21.97 |
| 50xxxx43 | male | 17 | 175 | 55.0 | 17.96 |
| 50xxxx14 | male | 67 | 175 | 88.0 | 28.73 |
| 50xxxx50 | female | 19 | 166 | 45.0 | 16.33 |
| 48xxxx43 | female | 14 | 162 | 36.5 | 13.91 |
| 45xxxx64 | male | 14 | 164 | 60.0 | 22.31 |
| 50xxxx83 | female | 28 | 166 | 54.0 | 19.60 |

**Supplementary Table 2:** Primer Information

| Primer | Sequence |
| --- | --- |
| Human MMP3-F | CCTACAAGGAGGCAGGCAAG |
| Human MMP3-R | CCCGTCACCTCCAATCCAAG |
| Human MMP13-F | TCGGCCACTCCTTAGGTCTT |
| Human MMP13-R | AAGTGGCTTTTGCCGGTGTA |
| Human ADAMTS4-F | GTCCCATGTGCAACGTCAAG |
| Human ADAMTS4-R | ATGCGGCCATCTTGTCATCT |
| Human ADAMTS5-F | GGGCACTGGCTACTATGTGG |
| Human ADAMTS5-R | CGTCACAGCCAGTTCTCACA |
| Human SOX9-F | GCTCTGGAGACTTCTGAACGA |
| Human SOX9-R | CCGTTCTTCACCGACTTCCT |
| Human Aggrecan-F | GTGCCTATCAGGACAAGGTCT |
| Human Aggrecan-R | GATGCCTTTCACCACGACTTC |
| Human Collagen II-F | ATGACAATCTGGCTCCCAAC |
| Human Collagen II-R | GAACCTGCTATTGCCCTC |
| Human β-actin-F | AGAGCTACGAGCTGCCTGAC |
| Human β-actin-R | AGCACTGTGTTGGCGTACAG |
| Human TLN2-F | AGCTCCTGGTCAGAGGACTT |
| Human TLN2-R | TTCCCGAATGACTCGACACG |
| Human THBS4-F | TTCCTGTAGAGCCTGTGGAGT |
| Human THBS4-R | GACCGAAGATGGTGGCTGAA |
| Human CHAD-F | AAGTTCAAGGGCCAGCACAT |
| Human CHAD-R | GGCTGGGTCAGAACCTGTTT |
| Human CHMP4B-F | CCCGAAACAGTCCCTCTACC |
| Human CHMP4B-R | TCTTCTTTCTTCTTGGCGGGT |
| Human RCN2-F | TGGCTTCTTCGATTTAATCTTCACT |
| Human RCN2-R | TCAAACCGGGACCTGAATCC |
| Human OTUB1-F | CAGCGACTCCGAAGGTGTTA |
| Human OTUB1-R | TTGCTGAATTCGGTCCTGCT |
| Human ABI3BP-F | GCAGCCTCAAACTGCAACAA |
| Human ABI3BP-R | GTTGCCCTTGGCTGTTCAAG |
| Human ARHGDIA-F | CGAGAGCCTGCGAAAGTACA |
| Human ARHGDIA-R | CCTTCAGCACAAACGACTGC |
| Human ATP6V0D2-F | CCCTCTATCCAACCTTCGGC |
| Human ATP6V0D2-R | ACGCTCGTAAAACACGTCCT |
| Human CCL2-F | CCCAAGCAGAAGTGGGTTCA |
| Human CCL2-R | GTGTCTGGGGAAAGCTAGGG |
| Human CCL5-F | GACAGCAAGTCTGGCAGGAT |
| Human CCL5-R | CACACTTGGCGGTTCTTTCG |
| Human ITGAL-F | AGTCACCCTGAGAGGTTCCA |
| Human ITGAL-R | AGCAGCAAACAAAATGCTTCCA |
| Human TNFSF11-F | GAAAGCAAATGGTGTGGCCG |
| Human TNFSF11-R | ACTGTGTTTTCACTGCGTGC |
| Rat MMP3-F | TGATGATGAACGATGGACAGATGATG |
| Rat MMP3-R | GCATTGGCTGAGTGAAAGAGACC |
| Rat MMP13-F | CCTTCCCTATGGTGATGATGATGATG |
| Rat MMP13-R | GCAAGAGTCACAGGATGGTAGTATG |
| Rat ADAMTS4-F | CGTTCCGCTCCTGTAACACTAAG |
| Rat ADAMTS4-R | AGGTCGGTTCGGTGGTTGTAG |
| Rat ADAMTS5-F | TCCTCTTGGTGGCTGACTCTTC |
| Rat ADAMTS5-R | CGGATGTGGTTCTCGATGCTTG |
| Rat SOX9-F | GGCGGAGGAAGTCGGTGAAG |
| Rat SOX9-R | AGATGGCGTTAGGAGAGATGTGAG |
| Rat Aggrecan-F | CACAGGCAGCACAGACACTTC |
| Rat Aggrecan-R | GGAGTCAAGGTCGCCAGAGG |
| Rat Collagen II-F | ACGCTCAAGTCGCTGAACAAC |
| Rat Collagen II-R | AATCCAGTAGTCTCCGCTCTTCC |
| Rat β-actin-F | TCGTGCGTGACATTAAAGAG |
| Rat β-actin-R | TGCCACAGGATTCCATACC |
| Rat TLN2-F | GGAGTGGCTGCGTCTACAAATG |
| Rat TLN2-R | CCTGCTTGGCTTCTTGGATGAG |
| Rat CCL2-F | AGGTCTCTGTCACGCTTCTGG |
| Rat CCL2-R | CAGTGAATGAGTAGCAGCAGGTG |

**Supplementary Table 3**

**Inverse variance weighted analysis**

| Exposures | SNPs | Inverse variance weighted | |
| --- | --- | --- | --- |
|  |  | OR (95%CI) | p |
| Job involves heavy manual or physical work | 19 | 1.0056 (1.0013-1.0099) | 0.0106 |
| Job involves mainly walking or standing | 12 | 1.0049 (1.0003-1.0094 ) | 0.0372 |

SNP, single-nucleotide polymorphism.

**Supplementary Table 4**

**Sensitivity, pleiotropy, and heterogeneity analyses**

| Exposures | Weighted Median | | MR-Egger | | Pleiotropy | | Heterogeneity | |
| --- | --- | --- | --- | --- | --- | --- | --- | --- |
|  | OR (95% CI) | p | OR (95% CI) | p | Intercept | p | Q | p |
| Job involves heavy manual or physical work | 1.0066 (1.0007-1.0125) | 0.0293 | 0.9891 (0.9963-1.0125) | 0.3713 | 0.0003 | 0.1770 | 19.9157 | 0.2786 |
| Job involves mainly walking or standing | 1.0038 (0.9982-1.0094) | 0.1866 | 0.9941 (0.9697-1.0191) | 0.6510 | 0.0002 | 0.4082 | 16.0824 | 0.0973 |

CI, confidence interval; MR, Mendelian randomisation; OR, odds ratio.

**
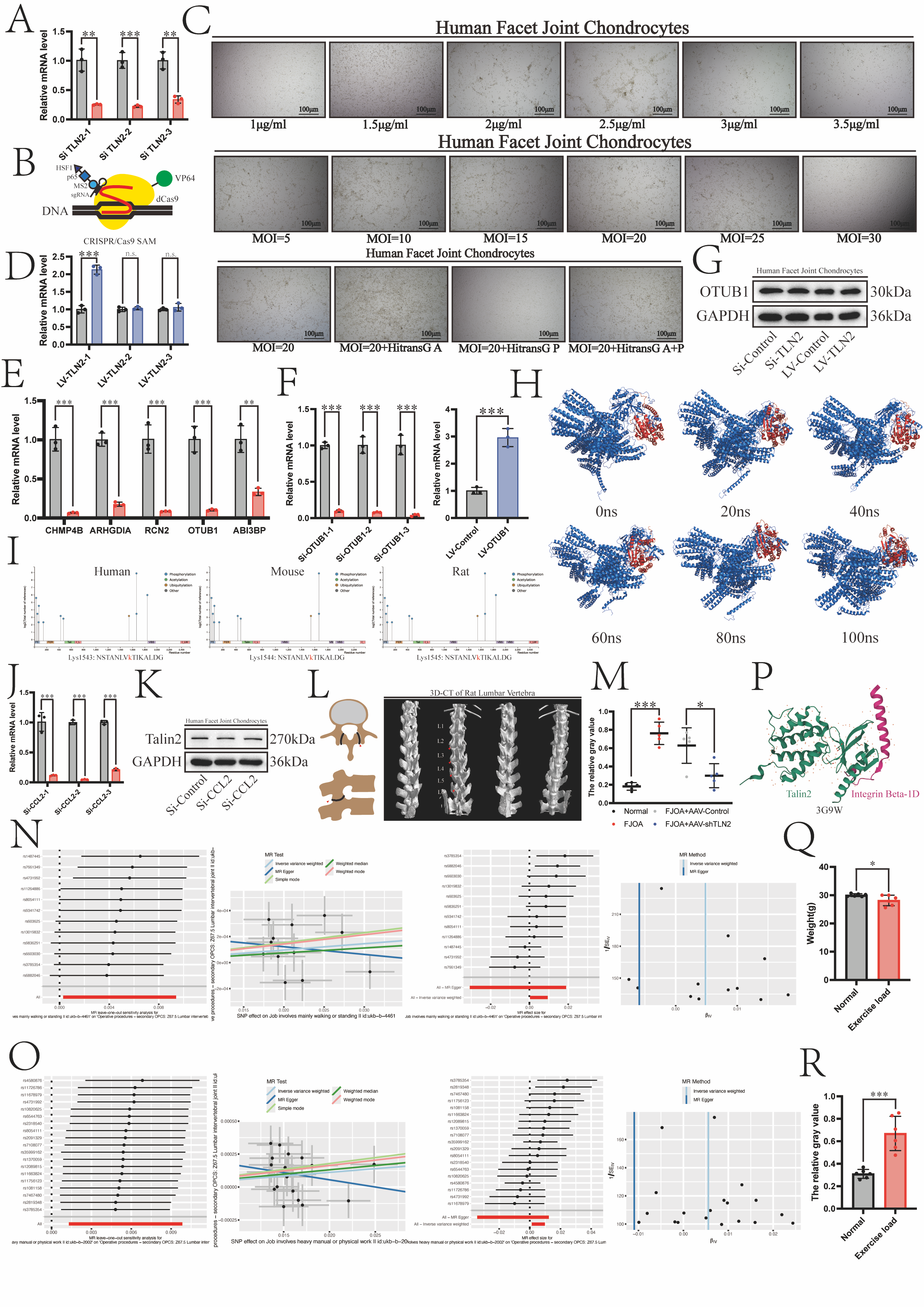
**

**Supplementary Figure. 1**:

**A** Efficiency of siRNA-mediated knockdown of *TLN2* determined by qRT-PCR. Values were normalized by β-actin mRNA levels. **B** Diagram of CRISPR/Cas9 synergistic activation mediator. **C** Tests of LV-TLN2 overexpression. Top, determination of the lowest lethal concentration of puromycin in chondrocytes. Middle, transfection of chondrocytes cultured in 2 μg/mL puromycin with LV-TLN2 to determine the lowest multiplicity of infection (MOI). Bottom, co-transfection of LV-TLN2 (MOI = 20) with HitransG-P and HitransG-A (Genechem, Shanghai, China) to determine optimal transfection characteristics. **D** Efficiency of TLN2 overexpression using different LV-TLN2 constructs determined by qRT-PCR. Values were normalized by β-actin mRNA levels. **E** Efficiency of siRNA-mediated knockdown of *CHMP4B*, *ARHGDIA*, *RCN2*, *OTUB1*, and *ABI3BP*. Values of respective mRNAs were normalized by β-actin mRNA levels. **F** Efficiency of siRNA-mediated knockdown and overexpression of *OTUB1* determined by qRT-PCR. Values were normalized by β-actin mRNA levels. **G** Western blot analysis of OTUB1 expression after the upregulation or downregulation of Talin2. **H** The spatial structure of Talin2/OTUB1 complex revealed by the molecular dynamic simulation at 0–100 ns. **I** Conserved the ubiquitination site of Talin2 in different species (human, Lys 1543; mouse, Lys 1544; rat, Lys 1545). **J** Efficiency of siRNA-mediated knockdown of *CCL2* determined by qRT-PCR. Values were normalized by β-actin mRNA levels. **K** Western blot analysis of Talin2 expression after the downregulation of *CCL2*. **L** Left, Anatomical location of the facet joint. Right, 3D-CT of rat lumbar spines (L1–L6). The red arrows represent the position of facet joints. **M** Statistical analysis of protein band grayscale values from different rat experimental groups. **N**, **O** Leave-one-out plots of “job involves mainly walking or standing” and “job involves heavy manual or physical work”. Mendelian randomisation (MR) results of the remaining IVs were calculated after removing the IVs one by one. Scatter plots of the causal relationship for the workload on lumbar intervertebral joint operation. The slope of each line corresponds to the estimated MR effect in different models. Detailed forest plots reveal the estimated MR effect of each IV in inverse variance weighted (IVW) models. Funnel plots assess the pleiotropy of observed causal associations for the workload on the lumbar intervertebral joint operation. **P** Interaction between Talin2 F2-F3 and integrin Beta-1D cytoplasmic tail (3G9W, PDB: https://doi.org/10.2210/pdb3G9W/pdb). **Q** Weights in the normal and exercise load groups. **R** Statistical analysis of protein band grayscale values from the normal and exercise load groups. Data are presented as the mean ± S.D. **p* < 0.05; ***p* < 0.01; ****p* < 0.001.
